# Analysis of Scyllo-Inositol in a Wistar Rat Animal Model—A Preliminary Study

**DOI:** 10.3390/ph18070954

**Published:** 2025-06-25

**Authors:** Karol Wiśniewski, Kamila Zglejc-Waszak, Tomasz Antonowski, Joanna Szablinska-Piernik, Jerzy Juskiewicz, Lesław Lahuta, Marcin Jozwik, Joanna Wojtkiewicz

**Affiliations:** 1 Students’ Scientific Club of Pathophysiologists, Department of Human Physiology and Pathophysiology, School of Medicine, University of Warmia and Mazury in Olsztyn, 10-082 Olsztyn, Poland; wisniewski.karol@op.pl (K.W.); t.antonowski@gmail.com (T.A.); 2 Department of Anatomy and Histology, School of Medicine, Collegium Medicum, University of Warmia and Mazury in Olsztyn, 10-085 Olsztyn, Poland; kamila.zglejc@uwm.edu.pl; 3 Department of Human Physiology and Pathophysiology, School of Medicine, Collegium Medicum, University of Warmia and Mazury in Olsztyn, 10-085 Olsztyn, Poland; 4 Department of Botany and Evolutionary Ecology, Faculty of Biology and Biotechnology, University of Warmia and Mazury in Olsztyn, 10-719 Olsztyn, Poland; joanna.szablinska@uwm.edu.pl; 5 Institute of Animal Reproduction and Food Research of Polish Academy of Sciences, 10-683 Olsztyn, Poland; j.juskiewicz@pan.olsztyn.pl; 6 Department of Plant Physiology, Genetics and Biotechnology, Faculty of Biology and Biotechnology, University of Warmia and Mazury in Olsztyn, 10-719 Olsztyn, Poland; lahuta@uwm.edu.pl; 7 Department of Gynecology and Obstetrics, School of Medicine, Collegium Medicum, University of Warmia and Mazury in Olsztyn, 10-045 Olsztyn, Poland; marcin.jozwik@uwm.edu.pl

**Keywords:** scyllo-inositol, cyclitols, pharmacokinetic, rat, Alzheimer

## Abstract

**Background**: Scyllo-inositol (SCI) is a naturally occurring carbocyclic sugar implicated in many biological processes. Studies have highlighted the potential of using SCI in Alzheimer’s therapy. However, in order to fully use this compound in the treatment of neurovegetative diseases, its pharmacokinetics must be thoroughly understood. **Objectives**: We undertook the task of analyzing SCI in a Wistar rat animal model. The aim of this study was to observe the changes in SCI concentration after oral administration. **Methods**: All rats received 10 mg/kg of SCI as a solution in distilled water by oral gavage. Estimated parameters were based on the serum concentration of SCI observed in six individual rats with regard to time. **Results**: The first peak concentration appeared at 30 min for SCI. Thereafter, the serum SCI concentration increased rapidly and reached its highest level after approximately 1.5 h. There was no second peak in SCI concentration. The elimination half-life was determined to be 10.07 h and the mean residence time was 14.52 h. There were no side effects of SCI supplementation noticed during the study. **Conclusions**: Although our results present an analysis of SCI immediately after oral administration up to 48 h, further studies are necessary.

## 1. Introduction

Cyclitols are a widespread group of naturally occurring compounds. From a chemical point of view, it is based on a cycloalkane chain that contains at least three hydroxyl groups, each attached to a different ring carbon. The most common cyclitols in eukaryotic cells are inositols (INSs) [1,2]. There are nine known forms of INS, namely scyllo-inositol (SCI), myo-inositol (MI), D-chiro-inositol, cis-inositol, epi-inositol muco-inositol, allo-inositol, L-chiro-inositol, and neo-inositol [3].

SCI and its derivates are widespread throughout biological systems. It has been identified in plants, such as legumes and cereals [4]. One of the largest concentrations of SCI (about 0.5 g/L) can be found in coconut milk. Worth mentioning is the fact that coconut milk has been successfully used as a growth-promoting agent in formulations of plant cell culture medium. High concentrations of SCI and MI are known to be one of the factors of its effectiveness [5]. Moreover, SCI can be found in mammalian tissues, especially in the brain, kidney, and lens tissue [3]. Furthermore, microorganisms such as *Bacillus subtilis* and *Rhizobium* species have been shown to produce SCI during INS metabolism, utilizing it as a carbon source under specific environmental conditions [6,7,8].

From a chemical point of view, SCI has a symmetrical structure in which all six hydroxyl groups are oriented equatorially. This confers chemical stability and unique physicochemical properties such as high aqueous solubility and low reactivity towards nucleophilic substitution [9,10]. SCI biosynthesis occurs predominantly through the enzymatic epimerization of MI [11,12]. Additionally, certain bacteria, including *Streptomyces griseus*, are capable of converting MI to SCI [13]. In synthetic chemistry, SCI is typically prepared from MI through a multi-step processes by a Mitsunobu reaction [10]. It can also be synthesized from para-benzoquinone via a conduritol intermediate [14]. Furthermore, there are reports that engineered microbial systems expressing inositol dehydrogenases can efficiently biosynthesize SCI from inexpensive substrates [15,16,17].

INSs are implicated in many biological processes including signal transduction, membrane biogenesis, phosphate storage, and membrane biogenesis. INS supplementation can be used in conjunction with other medications in the treatment of a variety of conditions including polycystic ovary syndrome, diabetes, metabolic syndrome, depression, psoriasis, and obsessive–compulsive disorder [2,3]. Furthermore, SCI has been shown to be a possible therapeutic option in Huntington’s [18] and Alzheimer’s disease [11,19]. There are also reports concerning the possible anticonvulsant properties of SCI [20,21] and its possible use in dementia treatment for patients with Down syndrome [22].

Literature analysis has shown that the most common and easiest method of INS supplementation appears to be oral [23]. MI, the dominant form of INSs in intracellular content, is almost fully (in 99.8%) absorbed in the human gastrointestinal tract. This uptake is possible due to active transport in a Na+-dependent manner [24]. Of note is the fact that sugars (for example, glucose) can significantly reduce MI uptake in a noncompetitive manner [3]. It is also known that MI can be converted to SCI by epimerases [11]. There are also reports showing that both SCI and MI have different concentrations in different tissues (for example, in the brain, their concentration is about 100-fold greater than in the surrounding tissues) [25,26]. It is also known that both SCI and MI can be transported by the same transporters [27,28]. Nevertheless, MI is mostly catabolized in the kidneys [29].

Although many studies have been conducted to identify the effects of INSs and their possible use in treating various conditions, there are not many reports concerning the pharmacokinetics (PK) of these compounds.

Antonowski and co-workers examined the PK of MI in Wistar rat models [30]. The study showed that the PK of MI can be described as a one-compartment model with first-order absorption and a zero-order endogenous input of checked inositol. The highest MI concentration was observed in the first hour after oral administration and then began to decrease. However, 24 h after administration, the MI level was still higher than before the supplementation. Worth mentioning is the fact that no serious side effects of MI administration were noticed during the study [30].

However, Liang and co-workers showed that the concentration of SCI changes in plasma, cerebrospinal fluid (CSF), and the brain in healthy young subjects. Moreover, a dose of 2000 mg twice a day was effective in elevating the SCI level in each tested tissue. The peak of SCI plasma concentration was reached at 3.8 h post-dose and then rapidly decreased until the next administration. The steady state of plasma concentration was achieved on day 5 of oral supplementation [31].

Nevertheless, the PK of SCI is still unclear, and further studies are needed to clarify the PK of SCI in animal models. Therefore, the aim of our study was an analysis of the changes in SCI concentration from the time of administration until 48 h after administration in the Wistar rat animal model.

## 2. Results

### 2.1. Determination of SCI in Serum

**Figure 1:**
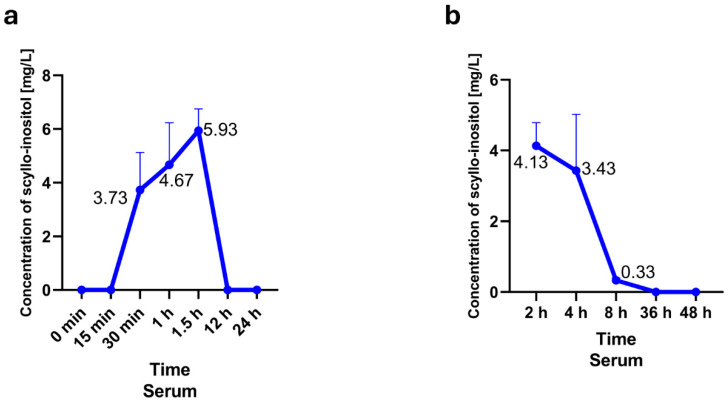
Summary of the obtained substance concentrations in blood in three rats: no. 1–3 (**a**) and no. 4–6 (**b**). (**a**) This graph shows the average concentration of SCI in serum over the studied period. The highest concentration was obtained during measurement 1.5 h after oral administration. (**b**) Summary of the obtained substance concentrations in blood in three rats: No. 4–6. This graph shows the average concentration of SCI in serum over the studied period. The highest concentration was obtained during measurement 2 h after oral administration. Data are expressed as means ± SEM.

During the analysis, we did not observe any additional peaks indicating any interference with the reading of results. Figure 1 shows the concentration of SCI in the studied rats at individual time points. In three rats (no. 1–3), the concentration of SCI was measured at the following time points: 0 min, 15 min, 30 min, 1 h, 1.5 h, 12 h, and 24 h. Nevertheless, in the remaining three rats (no. 4–6), the concentration of SCI was measured at the following time points: 2 h, 4 h, 8 h, 36 h, and 48 h (Table 1).

### 2.2. Pharmacokinetics

**Figure 2:**
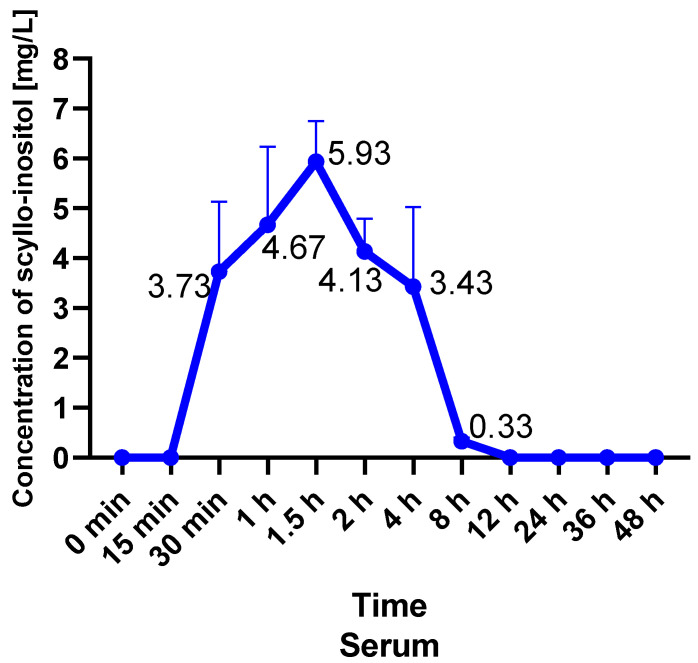
Mean serum concentration–time profiles of six rats after oral administration of SCI (3.92 mg). This graph shows the average concentration of SCI in serum over the studied period. The highest concentration was obtained during measurement 1.5 h after oral administration. Data are expressed as means ± SEM.

**Figure 3:**
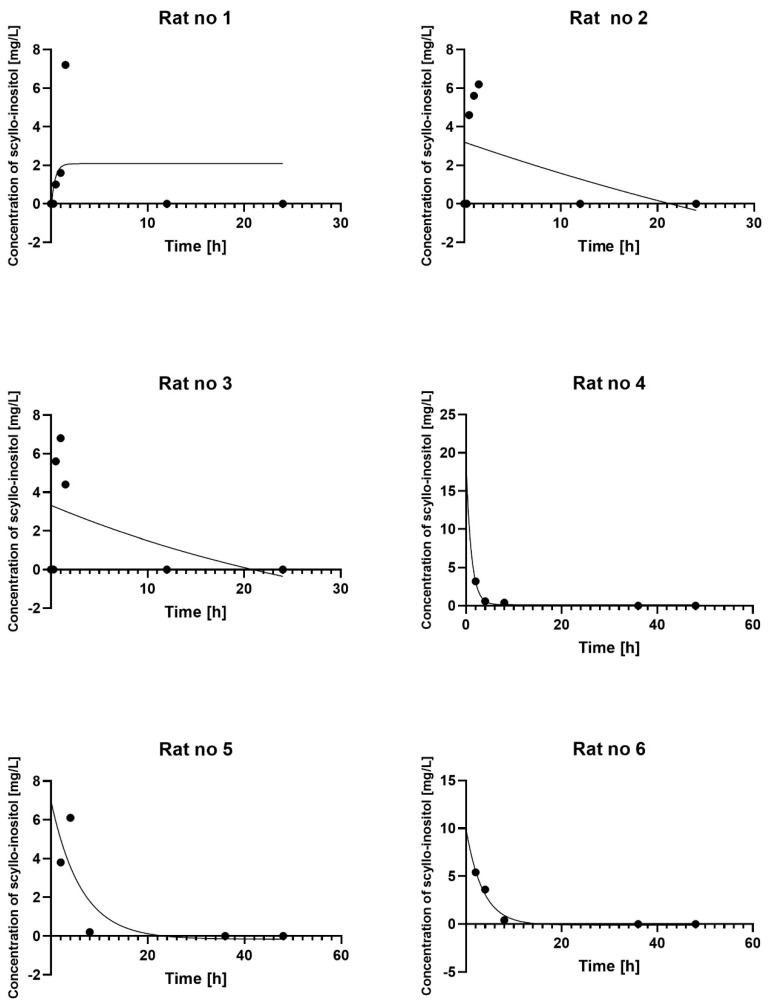
Plot of observed (circles) and individual predicted (solid lines) SCI concentration.

Estimated parameters were based on serum concentration–time profiles of SCI observed in six individual rats. Mean serum concentration–time profiles of SCI are shown in Figure 2, and the obtained parameters are shown in Table 2. PK data are presented for individual rats (Figure 3, Table 2).

After oral administration, SCI was absorbed slowly. At the point of administration, the concentration of SCI was 0 mg/L (below the limit of quantification). The first measurement of SCI concentration greater than 0 mg/L was recorded 30 min after administration (Figure 3, Table 2 and Table 3). The results indicate that SCI reaches its maximum concentration (Cmax) in the rat after 1.5 h. The mean Cmax was 5.93 mg/L (Figure 3, Table 2 and Table 3). There was no second peak of SCI concentration. The concentration of SCI decreased gradually to reach 4.13 mg/L two hours after administration and 3.43 mg/L four hours after administration. Then, the concentration of SCI decreased significantly; 8 h after administration, it was 0.33 mg/L (Figure 3, Table 3). At 12 h after administration, SCI concentration was 0 and remained at this level until the end of the study (Figure 3, Table 3). The Cmax and Tmax presented in Table 2 are the values calculated from each rat. The results presented in Table 3 are the values of the mean SCI dose per rat. Mean (±SEM) serum concentration–time profiles of 3.92 mg SCI are shown in Figure 2. The PK data presented in Table 3 represent the results based on Figure 2.

The total drug exposure integrated over time was 23.47 mg.hr/L (AUClast, area under the drug concentration–time curve from time zero to time of last quantifiable concentration, Table 3). The time that molecules of the dosed drug spent in the body was 14.52 h (MRT, Table 3). SCI was slowly eliminated from the body. The K value was 0.0689 h^−1^ and elimination half-life (T0.5e) was 10.07 h.

## 3. Discussion

SCI and its derivatives are naturally occurring substances of significance that have been emerging recently in health and biotechnology [32]. One of the disorders for which SCI has been tested was Alzheimer’s disease. SCI has been shown to decrease both the amount of insoluble amyloid proteins and amyloid plaque accumulation in the brain [33]. It has also been found to inhibit the binding of Aβ oligomers [34]. Moreover, mouse trials have shown positive effects after SCI administration [35]. However, clinical trials showed no benefits after SCI administration in patients with Alzheimer’s disease [19]. SCI has also been shown to be a possible therapeutic option in Huntington’s disease. In in vitro trials, it lowered the number of polyQ-Htt aggregates and decreased polyQ-Htt protein abundance [18]. Furthermore, SCI was found to inhibit the aggregation of α-synuclein into fibrils, a phenomenon implicated in Parkinson’s disease [36]. There are also reports about the possible anticonvulsant properties of SCI [20,21]. It is also worth mentioning that SCI has been used in dementia treatment for patients with Down syndrome. However, no significant positive impact of SCI administration was noticed [22].

PK is a branch of pharmacology dedicated to study the absorption, distribution, metabolism, and excretion of drugs and other various compounds in humans and animals [37]. PK parameters can be affected by many different factors, including compound properties, method of substance administration, dosage, and volume of distribution. Furthermore, other factors such as age, gender, and medical condition may play a role. The PK provides useful information about safety, efficiency, bioactive properties, metabolism, and possible toxicity on both animal and human models, which are crucial in the drug development process, as well as the treatment strategy [30,37,38,39].

Our study revealed that the first measurement of SCI concentration was recorded 30 min after administration. One-and-a-half hours after administration, SCI reached its maximum concentration. Nevertheless, we did not observe a second peak in SCI concentration. SCI concentrations were undetectable 12 h after oral administration, although the MRT was determined to be 14.52 h. Furthermore, the total drug exposure integrated over time was 23.47 mg.hr/L. There were no side effects of SCI supplementation noticed during the study.

Liang and co-workers [31] showed that the PK of SCI in eight healthy adult male subjects was tested. In this study, oral doses of 2000 mg of SCI were administrated two times a day for 10 days. After administration of the first dose, the plasma concentration of SCI rapidly increased. Plasma SCI reached its maximum (17.2 mg/mL) at 3.8 h post-dose and then declined rapidly until the next dose. The SCI concentration appeared to reach a steady state by day 5. After the last dose of SCI, a maximum concentration of 39.8 mg/mL was reached 2.9 h post-dose. Thereafter, similar to the profile observed after initial administration, plasma levels decreased rapidly. The AUC during each dose interval increased from 98.9 h mg/mL on day 1 to 277.0 h mg/mL on day 11, respectively. The increase in these parameters suggested an accumulation of SCI during the study. It is worth mentioning that the Cmax and AUC values of SCI in CSF increased from 6.5 mg/mL and 49.4 mg.hr/L on day 1 to 13.7 mg/mL and 153.0 mg.hr/L on day 11, respectively. This date also implicated the accumulation factor of SCI. Moreover, the brain concentration of SCI also increased (by 58–76%) during the study [31].

Liang et al. [31] in their study obtained a significantly higher maximum SCI concentration than we did. In our study, the Cmax obtained was 5.93 mg/L. Liang and co-workers [31] obtained a Cmax of 17,200 mg/L. However, the SCI dose used by Liang et al. [31] was significantly higher than ours. No side effects were reported in the aforementioned studies [31]. However, subsequent studies conducted by another team led to the withdrawal of the use of doses higher than 1000 mg per day [19].

In this study, we used a Wistar rat model, which is the most popular rat model. It is widely used in fields such as neuroscience, physiology, toxicology, and as a surgical model [40]. It is estimated that laboratory rats are the third most commonly used animal models in European research [41]. Only mice and fish are more often used. Nowadays, rats owe their popularity as a model organism to their widespread availability, short reproductive cycle, low breeding costs, and their ability to thrive in captive environments [40]. Wistar rats are more reliable compared to mice for certain studies (for example, addiction [42], cancer immunotherapy [43], and diabetes [44]).

Furthermore, Huang and co-workers analyzed the PK of scutellarin and its aglycone conjugated metabolites in male Sprague-Dawley rats. In this experiment, the PK after both intraperitoneal and oral supplementation was tested. Data revealed that the relative bioavailability of oral administration was very low compared to intraperitoneal injection [45].

Bioavailability studies in animal models are a critical step in evaluating how much of a substance is absorbed and becomes available in the body. A rat model is excellent to maximize the relevance of SCI to human health. Antonowski and co-workers [30] used a rat model for the PK analysis of MI. In this study, a dose of 20 mg/kg MI was orally administrated to rats and afterwards, the serum concentration of MI was measured [30]. The MI Cmax was observed in the first hour after administration in the serum of rats. Then, the concentration of MI began to decrease immediately. Nevertheless, after 24 h, the MI level was still higher than before the administration. The serum profile of the MI and SCI concentration was similar. However, AUC MI was lower than for SCI. Studies should be extended to include intraperitoneal injection or direct injection into blood vessels to determine the bioactivity of these INSs. It is worth mentioning that MI can be converted to SCI by epimerases [11].

Studies have indicated the simultaneous occurrence of MI and SCI in many tissues harvested from rats [46]. We can assume a close relationship and dependence in the metabolism of these INSs in the serum harvested from rats. We assume that two compounds or all INSs should be considered for their potential use in therapy against neurodegenerative diseases [46]. An interesting approach would be to analyze MI and SCI simultaneously, although it would be reasonable to assume that MI supplementation may increase SCI concentration [11].

Furthermore, studies have revealed that SCI may promote robust mutant Huntington protein degradation [18]. In this respect, the role of MI as a substrate for SCI conversion should also be taken into account. The latest studies have shown that MI, SCI, and other minor carbohydrates may be used as authenticity markers for the control of Italian bulk, concentrate, and rectified grape [47]. We see growing interest in the use of SCI for healthcare purposes, as well as in the biotechnology industry [47]. SCI is a rare but very valuable inositol [11]. SCI has shown potential in neurodegenerative disease therapy, broadly understood biotechnology, and for food authenticity control [46,47]. The use of microorganisms has enabled the cheap synthesis of SCI, making it an easily accessible compound for medicinal and food protection purposes [6,7,8,13]. Hence, PK analysis is an essential next tool for this compound’s use as a therapeutic. We have described PK in a Wistar rat animal model. We suggest that our results should be considered preliminary and validated in larger, more comprehensive studies.

## 4. Materials and Methods

### 4.1. Animals

All procedures were performed according to the Local Ethical Committee of Experiments on Animals guidelines in Olsztyn, Poland. The experiment was conducted in the same way as described by Antonowski et al. [30]. Briefly, the experiment was performed on male Wistar rats (n = 6). Rats were maintained in ventilated rooms under a standard day photoperiod (12:12 h light–dark) as well as with lights on from 700 to 1900 h at 21 °C. All rats had free access to food and water. At 42 days of age, rats were randomized into experimental groups (Table 1 and Table 4).

Before the administration of cyclitol (10 mg/kg) [20,21], the rats were fasting for 12 h (Table 1). After 2 h from the administration of the compound (SCI), the animals were given food. Access to water was unlimited during the experiments. The study group (n = 6) was divided into two subgroups (n = 3 rats per subgroup). This practice allows for the use of only 6 rats for the entire experiment in accordance with the 3 Rs principle. Rats received the compound at a dose of 10 mg/kg body weight. Rats were weighed before starting the compound administration. Cyclitol was administered to the rats from the study group using a stainless-steel gastric tube with a ball tip (manufacturer—Agnthos, curved gastric tube, 16G, 3 mm tip, 75 mm). Cyclitol was dissolved in distilled water to a maximum volume of 1 mL. The prepared solution was administered to the animal.

Blood samples were collected into Eppendorf tubes and were centrifuged at 2500× *g* for 10 min at 8 °C the following day. Blood was harvested from the tail at a specific time. Rats in the first subgroup (Rat nos. 1–3) had blood collected at 0 min, 15 min, 30 min, 1 h, 1.5 h, 12 h, and 24 h after oral SCI administration. Rats in the second subgroup (Rat nos. 4–6) had blood collected at 2 h, 4 h, 8 h, 36 h, and 48 h after SCI administration. Serum was stored at −80 °C for subsequent analyses.

### 4.2. Analysis of Scyllo-Inositol in Rat Serum

The analysis is described in detail by Antonowski and co-workers [30]. Briefly, the first step was to remove the protein, then concentrate the sample in a speed vacuum evaporator to dryness. Next, samples were analyzed using the high-resolution gas chromatography method. We used a gas chromatograph GC2010Plus (Shimadzu, Tokyo, Japan) with a capillary column ZEBRON ZB-1 (15 m length, 0.25 mm diameter, and 0.1 μm film thickness, Phenomenex, Torrance, CA, USA). Helium was used as a carrier gas (at a flow rate of 1.18 mL min^−1^). Superior technology that achieves high sensitivity and stability was used. By activating the new ecology mode feature whenever the system is in between analyses or after finishing a batch of analyses, the GCMS-QP2010 SE will use about 60% less carrier gas and 40% less power than previous models. Given typical operating conditions, this reduces helium gas consumption by about 2 cylinders (with a capacity of 7 m^3^) per year, reducing laboratory operating costs. Finally, scyllo-inositol was quantified by using a standard. The content of SCI in serum was calculated from the standard curves of the corresponding components [30].

### 4.3. Pharmacokinetic Analysis

The PK parameters were calculated using PK Solver. PKSolver is an add-in program for pharmacokinetic and pharmacodynamic data analysis from Microsoft [48]. As a noncompartment model with first-order absorption and zero-order endogenous input of checked inositol, we calculated the highest SCI concentration (Cmax) and the time of the highest compound concentration (Tmax). The area under the drug concentration–time curve from time zero to the time of the last quantifiable concentration (AUClast) was calculated using the trapezoidal rule. The volume over which the drug was distributed (Vd) and clearance (CL) were conducted by PK Solver. Clearance (CL) reflects the elimination of the drug from the body (CL = K*Vd; Vd = dose of SCI/K/AUClast). In addition, we calculated the terminal elimination half-life (T05e) after oral administration and residence time of SCI in the body (MRT). Nevertheless, we estimated K by log-linear regression analysis on data points on the terminal log-linear phase. Noncompartmental analysis is generally the preferred methodology to use in that it requires fewer assumptions than model-based approaches. We presented the results for individual rats, subgroups of rats, and for all subjects studied. The content of scyllo-inositol was calculated from the standard curves of appropriate components. To determine the terminal phase, 4–6 rats (3 rats) was sufficient.

### 4.4. Statistical Analysis

An analysis of all parameters was carried out with GraphPad Prism software (Version 10 Graph Pad Software Inc., La Jolla, CA, USA). Data are expressed as means ± SEM or individual data. Briefly, AUClast was calculated using the trapezoidal method. The T0.5 of oral administration was calculated as 0.693/K.

## 5. Conclusions

The PK results indicated that the progression of SCI is slow. The first measurement of SCI concentration was recorded 30 min after administration. The Cmax was reached after 1.5 h and was 5.93 mg/L. There was no second peak of scyllo-inositol concentration. T0.5e was determined to be 10.07 h and MRT was 14.52 h. There were no side effects of SCI supplementation noticed during the study. Studies revealed that SCI may have pleiotropic effects, which highlights its use in medicine. Nevertheless, further studies are needed to fully define the PK properties of SCI and its application in treating a wide range of disorders.

## Figures and Tables

**Table 1:** The list of concentrations of SCI in serum.

Time [h]	Concentration [mg/L]			Time [h]	Concentration [mg/L]		
	Rat No. 1	Rat No. 2	Rat No. 3		Rat No. 4	Rat No. 5	Rat No. 6
0	BLQ	BLQ	BLQ	2	3.2	3.8	5.4
0.25	BLQ	BLQ	BLQ	4	0.6	6.1	3.6
0.5	1	4.6	5.6	8	0.4	0.2	0.4
1	1.6	5.6	6.8	36	BLQ	BLQ	BLQ
1.5	7.2	6.2	4.4	48	BLQ	BLQ	BLQ
12	BLQ	BLQ	BLQ				
24	BLQ	BLQ	BLQ				

Abbreviations: BLQ—below the limit of quantification (0 mg/L).

**Table 2:** Summary table of pharmacokinetic (PK) parameters—single individuals. See also Figure 1 and Figure S2.

Rat No	Cmax [mg/L]	Tmax [h]	AUClast [mg.hr/L]	Vd [L]	CL [L/h]	K [h^−1^]	T0.5e [h]	MRT [h]
1	7.2	1.5	40.78	0.039	0.1	2.521	0.2749	0.397
2	6.2	1.5	38.63	7.93	0.11	0.01387	49.97	72.09
3	6.8	1	29.7	4.578	0.126	0.0276	25.14	36.26
Mean 1–3	6.733 ± 0.291 (95%CI: 7.984–5.483)	1.33 ± 0.167 (95%CI: 2.05–0.616)	36.37 ± 3.392 (95%CI: 50.966–21.774)	4.182 ± 2.287 (95%CI: 14.02–(−5.656))	0.112 ± 0.008 (95%CI: 0.145–0.079)	0.854 ± 0.833 (95%CI: 4.44–(−2.732))	25.128 ± 14.346 (95%CI: 86.853–(−36.596))	36.249 ±20.696 (95%CI: 125.297–(−52.799))
4	3.2	2	11.4	0.358	0.325	0.9102	0.7615	1.099
5	6.1	4	25.3	0.9967	0.16	0.161	4.306	6.212
6	5.4	2	22.6	0.566	0.162	0.2861	2.422	3.495
Mean 4–6	4.9 ± 0.874 (95%CI: 8.659–1.141)	2.667 ± 0.667 (95%CI: 5.535–(−0.202))	19.767 ± 4.255 (95%CI: 38.076–1.457)	7.985 ± 7.310 (95%CI: 39.437–(−23.467))	0.216 ± 0.055 (95%CI: 0.451–(−0.02))	0.452 ± 0.232 (95%CI: 1.449–(−0.545))	2.497 ± 1.024 (95%CI: 6.902–(1.909))	3.602 ± 1.477 (95%CI: 9.957–(−2.753))

Cmax—maximum concentration; Tmax—time to reach maximum concentration; AUClast—area under the drug concentration–time curve from time zero to time of last quantifiable concentration; CL—clearance; Vd—volume over which the drug is distributed; K—elimination rate constant; T0.5e—elimination half-life; MRT—mean residence time; ±SEM, 95%CI—confidence interval.

**Table 3:** Table of pharmacokinetic (PK) parameters of scyllo-inositol (SCI) after oral administration of 3.92 mg of SCI. See also Figure 2.

PK	Cmax [mg/L]	Tmax [h]	AUClast [mg.hr/L]	Vd [L]	CL [L/h]	K [h^−1^]	T0.5e [h]	MRT [h]
Scyllo-inositol 3.92 [mg]	5.93	1.5	23.47	2.43	0.167	0.0689	10.07	14.52

Cmax—maximum concentration; Tmax—time to reach maximum concentration; AUClast—area under the drug concentration–time curve from time zero to time of last quantifiable concentration; CL—clearance; Vd—volume over which the drug is distributed; K—elimination rate constant; T0.5e—elimination half-life; MRT—mean residence time.

**Table 4:** The list of body weights of animals used in the experiment and the doses of scyllo-inositol administered.

Rat No. and Weight	Dose of Scyllo-Inositol per Rat
1. 410.0 g	4.1 mg
2. 424.8 g	4.25 mg
3. 374.7 g	3.75 mg
Average weight (1–3): 403.17 g	Average dose (1–3): 4.033 mg
4. 371.1 g	3.71 mg
5. 405.7 g	4.06 mg
6. 366.2 g	3.66 mg
Average weight (4–6): 381 g	Average dose (4–6): 3.81 mg
Average weight (1–6): 392.08 g	Average dose (1–6): 3.92 mg

## Data Availability

The original contributions presented in the study are included in the article and Supplementary Materials, further inquiries can be directed to the corresponding author.

## Supplementary Materials

The following supporting information can be downloaded at: https://www.mdpi.com/article/10.3390/ph18070954/s1, Figure S1: Scyllo-inositol concentration in individual rats participating in the experiment (a,b), Figure S2: SCI concentration in individual rats participating in the experiment (a–f). In three rats (no. 1–3), the concentration of SCI was measured at the following time points: 0 min, 15 min, 30 min, 1 h, 1.5 h, 12 and 24 h. Nevertheless in the remaining three rats (no. 4–6), the concentration SCI was measured at the following time points: 2 h, 4 h, 8 h, 36 h and 48 h. See also Figure S1.

## Abbreviations

The following abbreviations are used in this manuscript:

SCI	Scyllo-inositol
MI	Myo-inositol
INS	Inositol
PK	Pharmacokinetics

## References

[1] Carlomagno G., Unfer V. (2011). Inositol safety: Clinical evidence. Eur. Rev. Med. Pharmacol..

[2] Antonowski T., Osowski A., Lahuta L., Górecki R., Rynkiewicz A., Wojtkiewicz J. (2019). Health-promoting properties of selected cyclitols for metabolic syndrome and diabetes. Nutrients.

[3] Bizzarri M., Fuso A., Dinicola S., Cucina A., Bevilacqua A. (2016). Pharmacodynamics and pharmacokinetics of inositol(s) in health and disease. Expert Opin. Drug Metab. Toxicol..

[4] Keller F., Ludlow M.M. (1993). Carbohydrate metabolism in drought-stressed leaves of pigeonpea (*Cajanus cajan*). J. Exp. Bot..

[5] George E.F., Hall M.A., Klerk G.-J.D. (2007). The components of Plant Tissue Culture Media II: Organic additions, osmotic and pH effects, and support systems. Plant Propag. Tissue Cult..

[6] Loewus F.A., Murthy P.P.N. (2000). *Myo*-inositol metabolism in plants. Plant Sci..

[7] Skøt L., Egsgaard H. (1984). Identification of ononitol and *O*-methyl-*scyllo*-inositol in pea root nodules. Planta.

[8] Turner B.L., Richardson A.E. (2004). Identification of *scyllo*-inositol phosphates in soil by solution phosphorus-31 nuclear magnetic resonance spectroscopy. Soil Sci. Soc. Am. J..

[9] Day G.M., van de Streek J., Bonnet A., Burley J.C., Jones W., Motherwell W.D. (2006). Polymorphism of *scyllo*-inositol: Joining crystal structure prediction with experiment to elucidate the structures of two polymorphs. Cryst. Growth Des..

[10] Chung S.-K., Kwon Y.-U., Chang Y.-T., Sohn K.-H., Shin J.-H., Park K.-H., Hong B.-J., Chung I.-H. (1999). Synthesis of all possible regioisomers of *scyllo*-inositol phosphate. Bioorganic Med. Chem..

[11] Thomas M.P., Mills S.J., Potter B.V. (2015). The “other” Inositols and their phosphates: Synthesis, biology, and medicine (with recent advances in *myo*-inositol chemistry). Angew. Chem. Int. Ed..

[12] Loewus F.A., Loewus M.W. (1983). *myo*-inositol: Its biosynthesis and metabolism. Annu. Rev. Plant Physiol..

[13] Bruton J., Horner W.H., Russ G.A. (1967). Biosynthesis of streptomycin. J. Biol. Chem..

[14] Podeschwa M., Plettenburg O., vom Brocke J., Block O., Adelt S., Altenbach H. (2003). Stereoselective synthesis of *myo*-, *neo*-, L-*chiro*, D-*chiro*-, *allo*-, *scyllo*-, and *epi*-inositol systems via conduritols prepared from p-benzoquinone. Eur. J. Org. Chem..

[15] Ramp P., Lehnert A., Matamouros S., Wirtz A., Baumgart M., Bott M. (2021). Metabolic Engineering of *Corynebacterium glutamicum* for production of *scyllo*-inositol, a drug candidate against Alzheimer’s disease. Metab. Eng..

[16] Yamaoka M., Osawa S., Morinaga T., Takenaka S., Yoshida K. (2011). A cell factory of *Bacillus subtilis* engineered for the simple bioconversion of *myo*-inositol to *scyllo*-inositol, a potential therapeutic agent for Alzheimer’s disease. Microb. Cell Factories.

[17] Morinaga T., Ashida H., Yoshida K. (2010). Identification of two *scyllo*-inositol dehydrogenases in *Bacillus subtilis*. Microbiology.

[18] Lai A.Y., Lan C.P., Hasan S., Brown M.E., McLaurin J. (2014). *Scyllo*-inositol promotes robust mutant huntingtin protein degradation. J. Biol. Chem..

[19] Salloway S., Sperling R., Keren R., Porsteinsson A.P., van Dyck C.H., Tariot P.N., Gilman S., Arnold D., Abushakra S., Hernandez C. (2011). A phase 2 randomized trial of ELND005, *scyllo*-inositol, in mild to moderate Alzheimer disease. Neurology.

[20] Nozadze M., Mikautadze E., Lepsveridze E., Mikeladze E., Kuchiashvili N., Kiguradze T., Kikvidze M., Solomonia R. (2011). Anticonvulsant activities of *myo*-inositol and *scyllo*-inositol on pentylenetetrazol induced seizures. Seizure.

[21] Wiśniewski K., Antonowski T., Juranek J., Podlasz P., Wojtkiewicz J. (2023). Antiepileptic properties of *scyllo*-inositol on pentylenetetrazol-induced seizures. Int. J. Mol. Sci..

[22] Rafii M.S., Skotko B.G., McDonough M.E., Pulsifer M., Evans C., Doran E., Muranevici G., Kesslak P., Abushakra S., Lott I.T. (2017). A randomized, double-blind, placebo-controlled, phase II study of Oral ELND005 (*scyllo*-inositol) in young adults with down syndrome without dementia. J. Alzheimer’s Dis..

[23] Facchinetti F., Bizzarri M., Benvenga S., D’Anna R., Lanzone A., Soulage C., Di Renzo G.C., Hod M., Cavalli P., Chiu T.T. (2015). Results from the International Consensus Conference on *myo*-inositol and D-*chiro*-inositol in obstetrics and gynecology: The link between metabolic syndrome and PCOS. Eur. J. Obstet. Gynecol. Reprod. Biol..

[24] Holub B.J. (1986). Metabolism and function of *myo*-inositol and inositol phospholipids. Annu. Rev. Nutr..

[25] Palmano K.P., Whiting P.H., Hawthorne J.N. (1977). Free and lipid *myo*-inositol in tissues from rats with acute and less severe streptozotocin-induced diabetes. Biochem. J..

[26] Michaelis T., Helms G., Merboldt K., Hänicke W., Bruhn H., Frahm J. (1993). Identification of *scyllo*-inositol in proton NMR spectra of human brain in vivo. NMR Biomed..

[27] Hager K., Hazama A., Kwon H.M., Loo D.D.F., Handler J.S., Wright E.M. (1995). Kinetics and specificity of the renal Na^+^/*myo*-inositol cotransporter expressed in *Xenopus* oocytes. J. Membr. Biol..

[28] Coady M.J., Wallendorff B., Gagnon D.G., Lapointe J.-Y. (2002). Identification of a novel Na^+^/*myo*-inositol cotransporter. J. Biol. Chem..

[29] Croze M.L., Soulage C.O. (2013). Potential role and therapeutic interests of *myo*-inositol in metabolic diseases. Biochimie.

[30] Antonowski T., Osowski A., Szczesny D., Szablińska-Piernik J., Juśkiewicz J., Lahuta L., Rynkiewicz A., Wojtkiewicz J. (2022). Pharmacokinetics of *myo*-inositol in a Wistar rat animal model. Int. J. Mol. Sci..

[31] Liang E., Garzone P., Cedarbaum J.M., Koller M., Tran T., Xu V., Ross B., Jhee S.S., Ereshefsky L., Pastrak A. (2013). Pharmacokinetic profile of orally administered *scyllo*-inositol (ELND005) in plasma, cerebrospinal fluid and brain, and corresponding effect on amyloid-beta in healthy subjects. Clin. Pharmacol. Drug Dev..

[32] Chatree S., Thongmaen N., Tantivejkul K., Sitticharoon C., Vucenik I. (2020). Role of inositols and inositol phosphates in energy metabolism. Molecules.

[33] Fenili D., Brown M., Rappaport R., McLaurin J. (2007). Properties of *scyllo*–inositol as a therapeutic treatment of ad-like pathology. J. Mol. Med..

[34] Jin M., Selkoe D.J. (2015). Systematic analysis of time-dependent neural effects of soluble amyloid β oligomers in culture and in vivo: Prevention by *scyllo*-Inositol. Neurobiol. Dis..

[35] McLaurin J., Kierstead M.E., Brown M.E., Hawkes C.A., Lambermon M.H., Phinney A.L., Darabie A.A., Cousins J.E., French J.E., Lan M.F. (2006). CYCLOHEXANEHEXOL inhibitors of AΒ aggregation prevent and reverse Alzheimer phenotype in a mouse model. Nat. Med..

[36] Ibrahim T., McLaurin J. (2016). A-synuclein aggregation, seeding and inhibition by *scyllo*-Inositol. Biochem. Biophys. Res. Commun..

[37] https://www.europeanreview.org/wp/wp-content/uploads/6.pdf.

[38] Hung W.-L., Chang W.-S., Lu W.-C., Wei G.-J., Wang Y., Ho C.-T., Hwang L.S. (2018). Pharmacokinetics, bioavailability, tissue distribution and excretion of Tangeretin in rat. J. Food Drug Anal..

[39] Nordberg M., Duffus J., Templeton D.M. (2004). Glossary of terms used in Toxicokinetics (IUPAC recommendations 2003). Pure Appl. Chem..

[40] Modlinska K., Pisula W. (2020). The norway rat, from an obnoxious pest to a laboratory pet. eLife.

[41] Directorate-General for Environment (European Commission) Report from the Commission to the European Parliament and the Council 2019 Report on the Statistics on the Use of Animals for Scientific Purposes in the Member States of the European Union in 2015–2017. https://op.europa.eu/en/publication-detail/-/publication/04a890d4-47ff-11ea-b81b-01aa75ed71a1.

[42] Vengeliene V., Bilbao A., Spanagel R. (2014). The alcohol deprivation effect model for studying relapse behavior: A comparison between rats and mice. Alcohol.

[43] Bergman I., Basse P.H., Barmada M.A., Griffin J.A., Cheung N.-K.V. (2000). Comparison of in vitro antibody-targeted cytotoxicity using mouse, rat and human effectors. Cancer Immunol. Immunother..

[44] Obrosova I.G., Drel V.R., Kumagai A.K., Szábo C., Pacher P., Stevens M.J. (2006). Early diabetes-induced biochemical changes in the retina: Comparison of rat and mouse models. Diabetologia.

[45] Huang J.M., Weng W.Y., Huang X.B., Ji Y.H., Chen E. (2005). Pharmacokinetics of scutellarin and its aglycone conjugated metabolites in rats. Eur. J. Drug Metab. Pharmacokinet..

[46] Ma K., Thomason L.A., McLaurin J. (2012). scyllo-Inositol, preclinical, and clinical data for Alzheimer’s disease. Adv. Pharmacol..

[47] Paolini M., Perini M., Allari L., Tonidandel L., Finato F., Guardini K., Larcher R. (2023). *Myo*-Inositol, *Scyllo*-Inositol, and Other Minor Carbohydrates as Authenticity Markers for the Control of Italian Bulk, Concentrate, and Rectified Grape Must. Molecules.

[48] Zhang Y., Huo M., Zhou J., Xie S. (2010). PKSolver: An add-in program for pharmacokinetic and pharmacodynamic data analysis in Microsoft Excel. Comput. Methods Programs Biomed..

