# Supplementary material for: Analysis of Scyllo-Inositol in a Wistar Rat Animal Model—A Preliminary Study
Source: Pharmaceuticals (Basel). 2025 Jun 25;18(7):954. doi: 10.3390/ph18070954 (PMC12299890; doi:10.3390/ph18070954)
Supplement: Supplementary file 1 [file pharmaceuticals-18-00954-s001.zip › pharmaceuticals-3708127-supplementary.pdf]

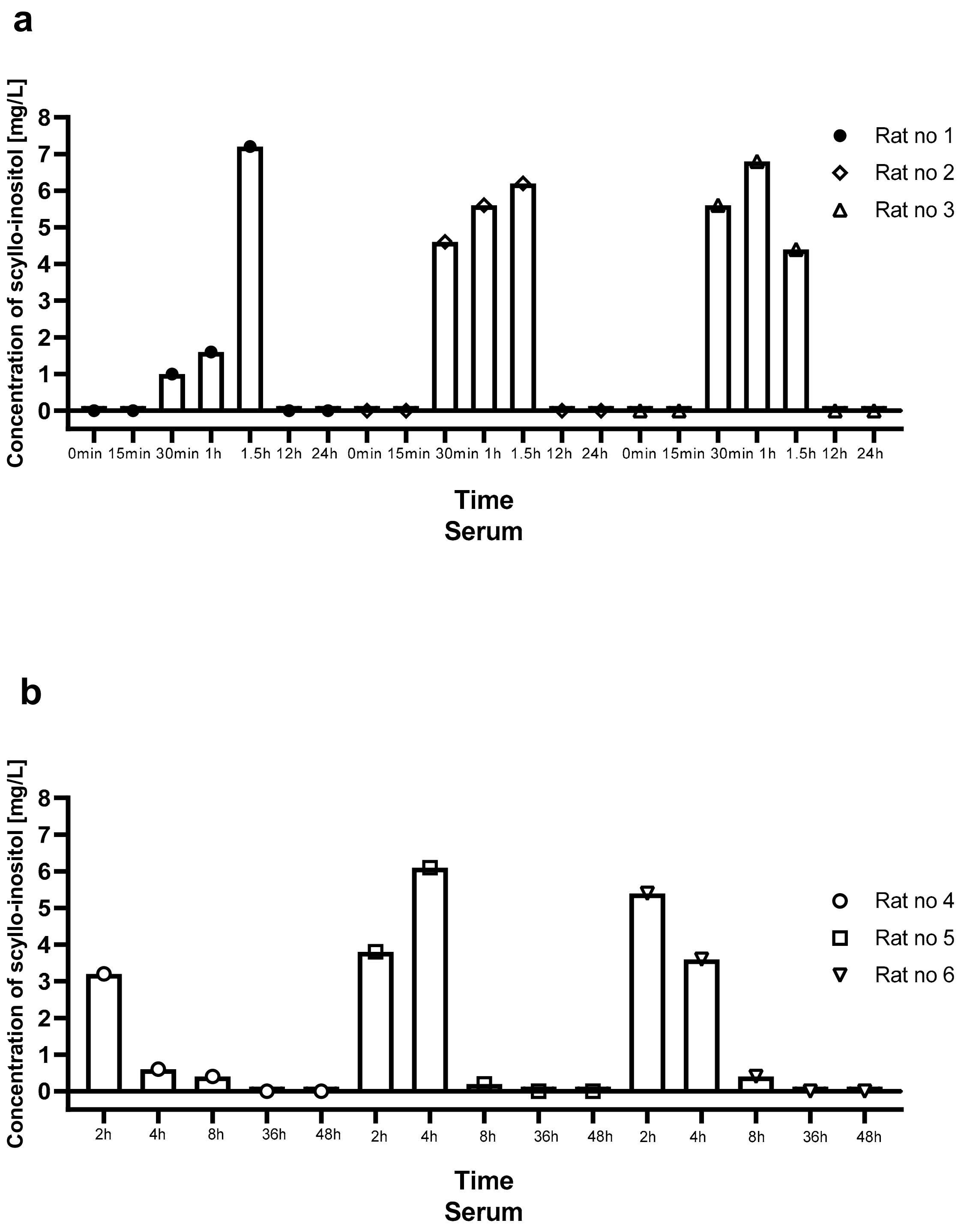

Figure S 1. Scyllo-inositol concentration in individual rats participating in the experiment (a-b). The concentration of scyllo-inositol at the studied time points was similar between individuals.

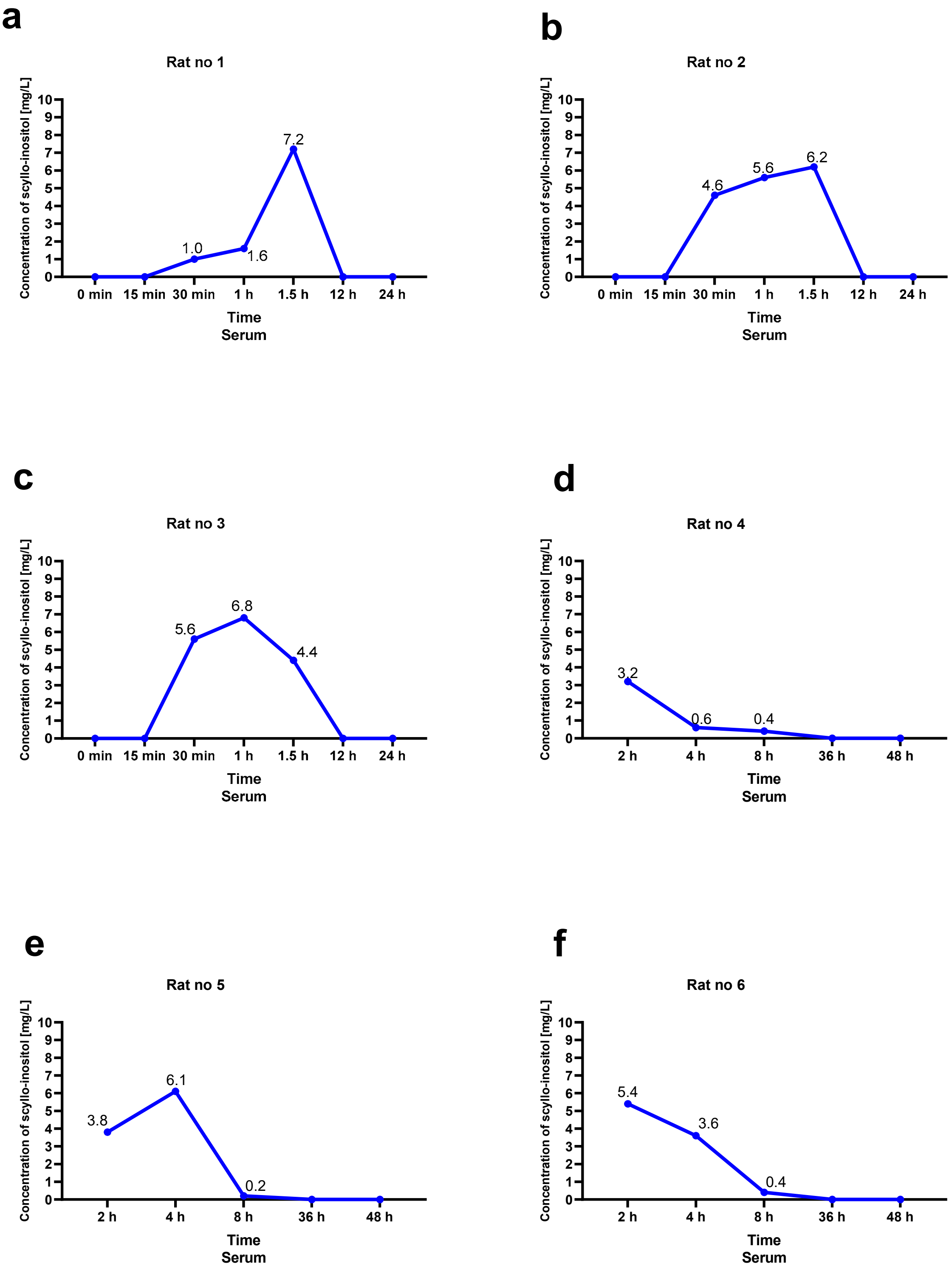

Figure S2. SCI concentration in individual rats participating in the experiment (a-f). In three rats (nos. 1-3), the concentration of SCI was measured at the following time points: 0 min, 15 min, 30 min, 1 h, 1.5 h, 12 h, and 24 h. Nevertheless, in the remaining three rats (nos. 4-6), the concentration of SCI was measured at the following time points: 2 h, 4 h, 8 h, 36 h, and 48 h. See also Figure S 1.
